# The Role of MicroRNAs as Predictors of Response to Tamoxifen Treatment in Breast Cancer Patients

**DOI:** 10.3390/ijms161024243

**Published:** 2015-10-14

**Authors:** Nina G. Egeland, Siri Lunde, Kristin Jonsdottir, Tone H. Lende, Deirdre Cronin-Fenton, Bjørnar Gilje, Emiel A. M. Janssen, Håvard Søiland

**Affiliations:** 1 Department of Pathology, Stavanger University Hospital, Gerd Ragna Bloch Thorsens Gate 8, 4011 Stavanger, Norway; E-Mails: kristin.jonsdottir@sus.no (K.J.); emilius.adrianus.maria.janssen@sus.no (E.A.M.J.); 2 Department of Mathematics and Natural Sciences, University of Stavanger, 4036 Stavanger, Norway; 3 Department of Breast and Endocrine Surgery, Stavanger University Hospital, 4011 Stavanger, Norway; E-Mails: tone.hoel.lende@sus.no (T.H.L.); havard.soiland@sus.no (H.S.); 4 Department of Clinical Science, University of Bergen, Postboks 7804, 5020 Bergen, Norway; 5 Department of Clinical Epidemiology, Aarhus University, Science Center Skejby, Olof Palmes Allé 43, Aarhus N, 8200 Aarhus, Denmark; E-Mail: dc@clin.au.dk; 6 Department of Haematology and Oncology, Stavanger University Hospital, Gerd Ragna Bloch Thorsens Gate 8, 4011 Stavanger, Norway; E-Mail: bjornar.gilje@sus.no

**Keywords:** breast cancer, tamoxifen, endocrine resistance, microRNA, biomarker

## Abstract

Endocrine therapy is a key treatment strategy to control or eradicate hormone-responsive breast cancer. However, resistance to endocrine therapy leads to breast cancer relapse. The recent extension of adjuvant tamoxifen treatment up to 10 years actualizes the need for identifying biological markers that may be used to monitor predictors of treatment response. MicroRNAs are promising biomarkers that may fill the gap between preclinical knowledge and clinical observations regarding endocrine resistance. MicroRNAs regulate gene expression by posttranscriptional repression or degradation of mRNA, most often leading to gene silencing. MicroRNAs have been identified directly in the primary tumor, but also in the circulation of breast cancer patients. The few available studies investigating microRNA in patients suggest that seven microRNAs (miR-10a, miR-26, miR-30c, miR-126a, miR-210, miR-342 and miR-519a) play a role in tamoxifen resistance. Ingenuity Pathway Analysis (IPA) reveals that these seven microRNAs interact more readily with estrogen receptor (ER)-independent pathways than ER-related signaling pathways. Some of these pathways are targetable (e.g., PIK3CA), suggesting that microRNAs as biomarkers of endocrine resistance may have clinical value. Validation of the role of these candidate microRNAs in large prospective studies is warranted.

## 1. Introduction

Breast cancer is a heterogenic disease that demands an individualized treatment plan, incorporating both patient and tumor information. The development of breast cancer is a highly complicated biological process in which the alteration of women’s physiology and the hormonal status plays a significant role [1]. The biological profile of breast cancer differs between the very young (<45 years) and elderly patients (>70 years). Tumors of younger patients are more often Estrogen Receptor alpha (ER) negative (30% are ER−) with higher average proliferation (Mitotic Activity Index (MAI) = 12.8), while elderly patients more often present with ER positive tumors (90% are ER+) and a much lower average proliferation (MAI = 8.7) [2]. Currently, biomarkers such as ER, Progesterone Receptor (PgR) and the Human Epidermal growth factor-like Receptor 2 (HER2) expression level, as well as proliferation status as measured by Ki-67, roughly distinguish patients according to breast cancer subtypes and help inform treatment choice [3,4]. These biomarkers represent important biological processes in the development and progression of breast cancer. In addition to these biological factors, clinical characteristics including the extent of cancer spread, tumor size, lymph node involvement, and evidence of any metastases (TNM), are used to determine the most effective treatment course. For most breast cancer patients, surgical removal of the tumor is primary treatment. In addition, depending on the specific characteristics of the individual tumor, adjuvant therapy comprising systemic treatment with chemotherapy, endocrine therapy, anti-HER2 treatment and/or zoledronic acid, and postoperative radiation therapy are recommended to reduce the risk of relapse [5].

Despite the effectiveness of surgery and extensive adjuvant treatments in breast cancer, challenges concerning over- and under-treatment and recurrence prediction persist. Over-treatment can induce temporary or chronic side effects, significantly lowering quality of life. On the other hand, under-treatment can lead to disease recurrence and metastasis, almost always with life-threatening consequences.

Two-thirds of breast cancer patients have ER+ tumors and are candidates for endocrine therapy [6,7]. Tamoxifen is recommended for premenopausal women, in whom aromatase inhibitors (AIs) are contraindicated [8], whereas AIs are the treatment of choice for postmenopausal women. Still, tamoxifen is an alternative or sequential treatment for postmenopausal patients, depending on their risk of tamoxifen side effects [9,10]. Endocrine therapy reduces the five-year recurrence risk by about one-half [6]. However, patients with identical prognostic factors at diagnosis can vary substantially in their clinical course and treatment response. Endocrine therapy resistance can either exist from the start of diagnosis (*de novo*/intrinsic resistance) or develops during the course of treatment (acquired resistance) [11]. Unfortunately, resistance to therapy as well as over- and under-treatment, are difficult to foresee with the current biomarkers. Thus, acquired resistance is hard to predict before a local or systemic relapse has occurred and becomes clinically overt. Therefore, improved prognostic and predictive biomarkers (measured in the primary tumor), as well as biomarkers for monitoring drug response (measured in blood) are urgently needed.

While many new biomarkers have been described over the last decades very few have made it from the laboratory into the clinic. Pultz *et al.* recently reviewed several biomarkers from relevant literature and sorted them according to their potential clinical relevance. They suggested that 15 of these markers should be validated in the clinic; amongst which microRNA was mentioned [12]. MicroRNAs are a short form of non-coding single-stranded RNA about 22 nucleotides in length. MicroRNAs regulate gene expression by posttranscriptional repression or degradation of mRNA, most often leading to gene silencing [13].

Although recently discovered, a great body of evidence is accumulating implying that miRNAs might provide both predictive and prognostic potential as biomarkers. In this review, we discuss the potential clinical utility of microRNAs as determinants of tamoxifen resistance in breast cancer patients, and how and where they interact with biological pathways in order to mediate such tamoxifen resistance.

### 1.1. Estradiol and the Estrogen Receptor

**Figure 1:**
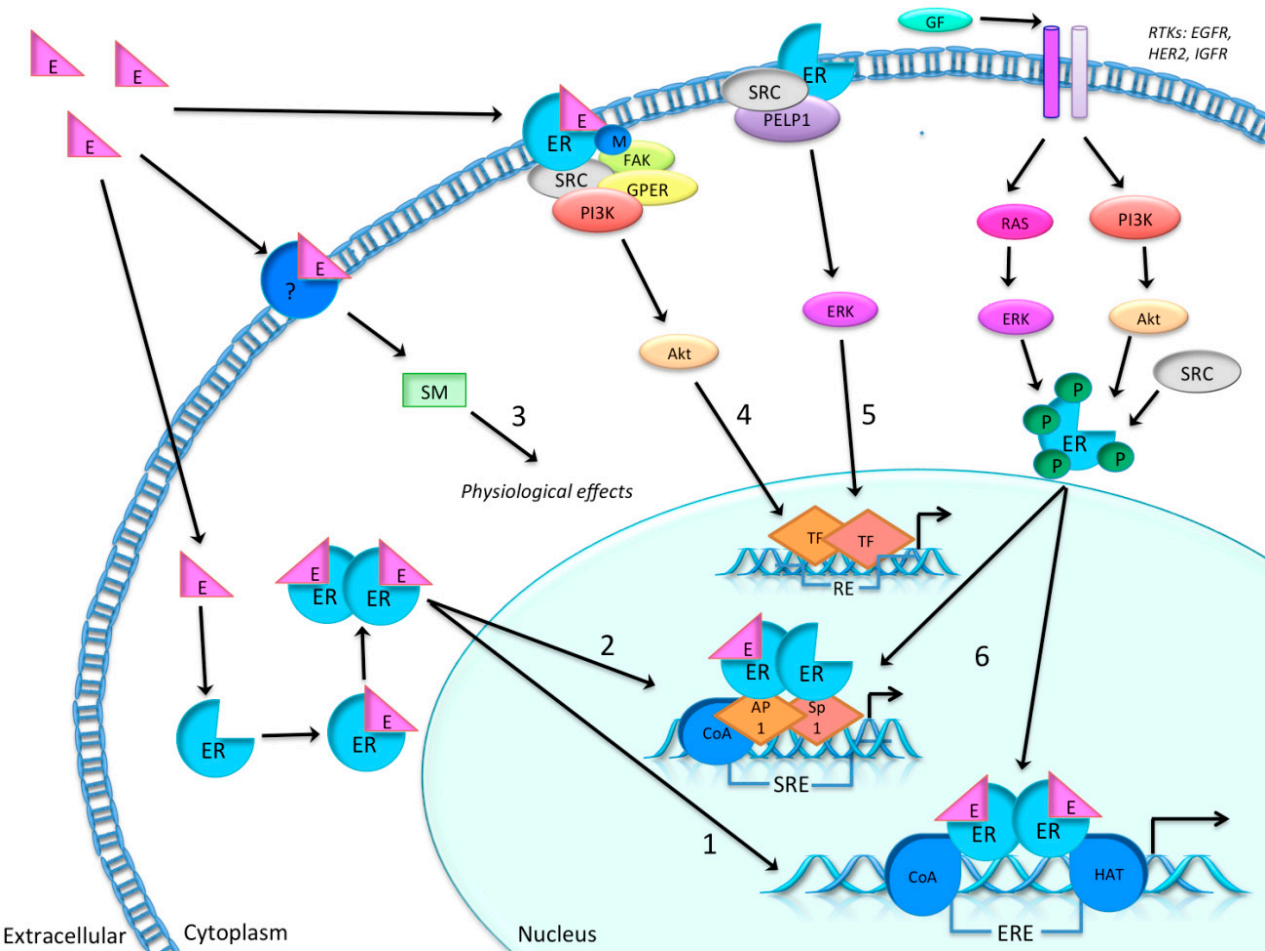
Simplified possible molecular signaling pathways (**1** to **6**) of estrogen (E) and estrogen receptors (ER). (**1**) Classical and direct pathway: ligand activation is followed by binding to the estrogen response element (ERE), including coactivators (CoA) and histone acetyl transferases (HATs) before gene regulation is modified; (**2**) tethered pathway: ligand dependent pathway which includes protein-protein interaction with other transcription factors, e.g., activator protein 1 (Ap1) and specificity protein 1 (Sp1), after ligand activation, thereby regulating genes by indirect DNA binding following serum response element (SRE) activation of transcription; (**3**) non-genomic ligand dependent reaction: the receptor (e.g., classical ER, ER isoform or other receptors) is activated by a ligand, which may be associated with the membrane. This is then followed by signaling cascades initiated by second messengers (SM), initiating a rapid physiological response, which does not involve gene regulation; (**4**) ligand-dependent reaction: ER is methylated by ligand induction and ER–phosphoinositide 3-kinase (PI3K)–steroid receptor coactivator (SRC)-focal adhesion kinase (FAK) forms a complex that further activates the serine/threonine–protein kinase Akt, which then activates transcription without ER binding to DNA; (**5**) ligand independent reaction: ER–SRC–proline-, glutamic acid and leucine-rich protein 1 (PELP1) forms a complex which then activates transcription, also without ER binding to DNA; (**6**) another ligand independent reaction activates through other signaling pathways, like growth factor signaling by downstream events of receptor tyrosine kinase (RTKs), such as epidermal growth factor receptor (EGFR), human epidermal growth factor receptor 2 (HER2) and the insulin-like growth factor receptor (IGFR) [11,23].

As illustrated in Figure 1, the biological activities of estrogens are mediated by ERs, which upon activation by cognate ligands form homodimers, or heterodimers with other ER-ligand complexes [14,15], and activate transcription of specific genes containing the estrogen response element (ERE) [16,17].

### 1.2. Endocrine Treatment Regimens

**Figure 2:**
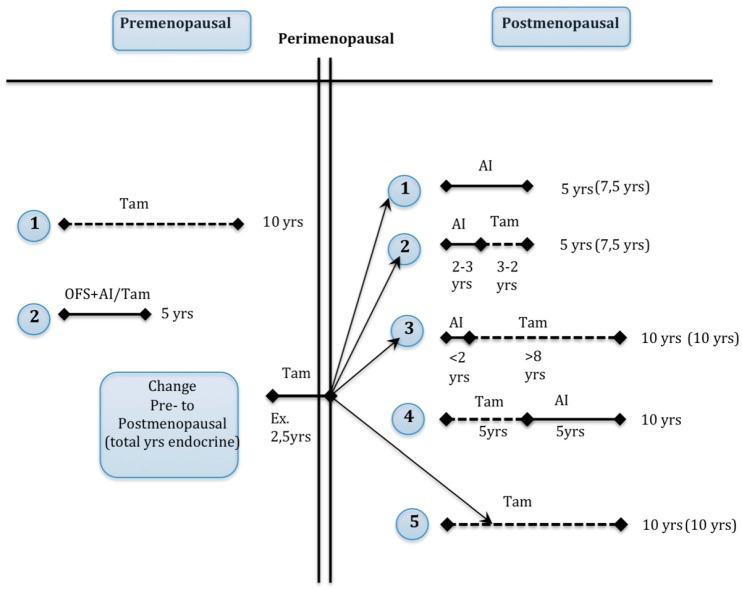
Overview of the adjuvant endocrine treatment guidelines for ER+ breast cancer patients according to the Norwegian Breast Cancer Group (NBCG) 2015 [24], and based on international recommendations (St. Gallen, 2013). There are two options for premenopausal patients (1 and 2 on the left side) and five options for postmenopausal patients (1–5 on the right side) comprising aromatase inhibitor (AI), tamoxifen and ovarian function suppression (OFS) alone or in combination. Total duration of endocrine treatment for a premenopausal patient that becomes postmenopausal after two or five years on tamoxifen (example) is illustrated in brackets. The choice between alternatives 1–5 is made individually based upon tumor biology, side effects and preferences among clinicians and patients. Peri: perimenopausal; TAM: tamoxifen; Yrs: years; Dotted line: years on tamoxifen; Solid line: years on AI.

Endocrine treatment regimens for breast cancer patients comprise a dual strategy by either blocking the estrogen action at the ER-level (tamoxifen), or by inhibition of the *in vivo* estrogen synthesis in the whole body. In postmenopausal patients the latter is achieved by AIs alone, while pre-menopausal women need ovarian function suppression (OFS) and AIs in concert. The current treatment regimens for pre-, peri- and postmenopausal ER+ breast cancer women in Norway are illustrated in Figure 2. These national guidelines are based on international recommendations [3], and are similar to the guidelines of the National Comprehensive Cancer Network [5]. Note that endocrine treatment has been extended to 10 years of treatment as a result of recent publications [7,18].

Tamoxifen is a selective ER modulator (SERM) and the most frequently used anti-estrogen adjuvant treatment for ER+ pre-menopausal women. Tamoxifen is also a standard endocrine therapy for treatment of postmenopausal women with breast cancer, although AIs are more frequently used (see Figure 2 above). Depending upon the tissue, tamoxifen may function as an agonist or antagonist, recruiting either coactivators or corepressors to the ER transcription complex [19]. Tamoxifen exhibits antagonistic effects in breast tissue, thus has preventive effects on breast cancer development [20] and cytotoxic effects on breast cancer cells [21]. Tamoxifen also exerts agonistic effects in the uterus, increasing the risk of endometrial hyperplasia and malignancy [22].

The tamoxifen metabolic pathway is complex. In general, tamoxifen is oxidized in the liver by phase I metabolism involving various enzymes encoded by polymorphic genes including cytochrome P450 2D6 (CYP2D6) into two active metabolites: 4-hydroxy-*N*-desmethyl tamoxifen (endoxifen) and 4-hydroxytamoxifen (4-OHT). Endoxifen and 4-OHT then undergo phase II conjugation reactions and further find their way into the cancer cells. In breast cancer cells, 4-OHT acts as an antagonist preventing estrogen from binding to the ER, thus preventing proliferation and cell growth [25,26]. Levels of estrogen have been correlated with tamoxifen metabolite concentration in serum [27,28]. Due to polymorphic metabolic enzymes in the tamoxifen pathway, there are inter-individual differences in the concentration of the active metabolites in serum and therefore a potential for variation in drug effectiveness [29]. The serum concentration of tamoxifen and its metabolites increases with age, which could explain part of the inter-patient variation of active metabolite concentration in serum [30].

In postmenopausal patients, peripheral conversion of androgens into estrogens takes place in various tissues [31]. Third generation AIs cause more than 98% inhibition of this extra-ovarian aromatase activity and create extremely low serum and tissue levels of estrogens [32,33]. The systemic hypo-estrogenic state produced by AIs may explain their superiority to tamoxifen when administered upfront adjuvantly in postmenopausal patients [34,35]. However, this beneficial difference in survival disappears after two years of AI treatment [35], and therefore tamoxifen treatment for at least three years might also follow in postmenopausal patients [24] (Figure 2).

### 1.3. Resistance to Tamoxifen

**Figure 3:**
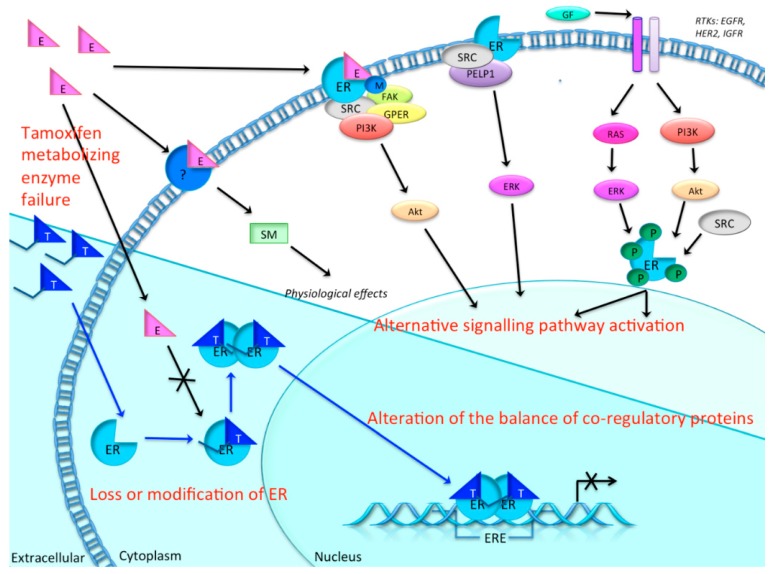
The tamoxifen pathway and possible mechanisms of endocrine resistance in breast cancer cells. Prior to entering the breast cancer cell, tamoxifen (T) is metabolized in the liver into the two active metabolites, endoxifen and 4-hydroxytamoxifen (4-OHT). When these metabolites enter the cell (blue background) they can bind to estrogen receptors (ERs), thereby blocking the binding of estrogen. ERs bound to tamoxifen then dimerize, enter the nucleus and bind to estrogen response element (ERE). However, the necessary coactivators will not be recruited by the ER–tamoxifen complex. Only corepressors are recruited, therefore gene transcription is not activated. In tamoxifen resistance, this blocking is compromised due to several possible mechanisms: e.g., changes in activity of the metabolizing enzymes of tamoxifen, loss or modification of ER expression, alternative signaling pathways for proliferation and growth, and alterations in the balance of co-regulatory proteins and altered expression of microRNAs [37,39]. Black arrow: normal estrogen pathway. Blue arrow: tamoxifen pathway. Crossed arrow: disrupted pathway.

In approximately 30% of ER+ breast cancer patients, endocrine treatment fails due to tamoxifen resistance [36]. As illustrated in Figure 3, mechanisms of tamoxifen resistance may involve changes in the activity of enzymes that metabolize tamoxifen, loss or modification of ER expression, alterations in the balance of co-regulatory proteins, altered expression of specific microRNAs, or the activation of alternative signal transduction pathways that can further promote tumor growth [37,38]. Regardless, it is likely that the pathways involved in *de novo versus* acquired resistance are different [39]. Therefore, monitoring the development of resistance to tamoxifen and the exploration of new therapeutic targets is pivotal.

### 1.4. MicroRNAs

MicroRNAs are defined as short non-protein-coding RNA molecules, of which the mature form is about 22 nucleotides in length. Each microRNA is complementary or partially complementary to one or more mRNA molecules, and its main function is to post-transcriptionally down-regulate gene expression by either binding directly to its mRNA target, or by cleaving target mRNA by binding to its 3′-UTR region. According to the microRNA database miRBase v.21, more than 2603 human microRNAs have been identified so far [40]. A single microRNA can potentially target up to 200 mRNAs; and the same mRNA molecule may also be targeted by different microRNAs [41,42], underlining the wide range and complexity of their functions. MicroRNAs have been shown to play a pivotal role in numerous biological processes, cellular pathways and networks. Many major cellular functions such as development, differentiation, growth, metabolism, survival, motility and proliferation are, in part, regulated by microRNAs. Since the link between cancer and microRNAs was first demonstrated in 2002 [43], microRNAs have also been shown to be involved in multiple cancer types, and microRNA-encoding genes are often located at genomic regions known to be associated with cancer [44]. More specifically, microRNAs are often involved in mechanisms underlying tumorigenesis and tumor progression, where they act as either tumor suppressor microRNAs or as tumor-promoting microRNAs [45].

## 2. Methods

### 2.1. Literature Search

To find relevant original articles for tamoxifen related microRNA, we performed a search in PubMed on the first of July 2015 using the words “microRNA” and “tamoxifen” (89 articles), filtering for human species (53 articles). Further selection, excluding review articles and focusing on studies that included patient material only, resulted in six studies [46,47,48,49,50,51] (see Table 1).

**Table 1:** Candidate microRNAs involved in tamoxifen response.

MicroRNA	Material and Patients	Clinical Outcome	Reference	Predicted Targets or Pathways
miR-342-5p	FFPE of tamoxifen-treated primary tumor, *n* = 16	Ten patients responded to tamoxifen and had non-recurrent disease: two-fold the level of miR-342 expression; Six patients developed recurrences and metastasis during tamoxifen treatment and had low levels of miR-342	[46]	Target genes: *GEMIN4* and *BMP7* Predicted pathways: cell death, apoptosis of breast cancer cells, mitotic roles of polo-like kinase
miR-30c-5p	Primary tumors, tamoxifen-treated following advanced disease, *n* = 246	Increasing levels of miR-30c was associated with clinical benefit of tamoxifen treatment, as measured by longer PFS	[49]	Target genes: *PPARGC1B*, *Makorin-3*, *UBAC1*, *PTPDC1* Predicted pathways*:* HER2, signal ransduction, and oncology pathway, RAC1 cell motility signaling pathway
miR-210-3p	Fresh-frozen tamoxifen-treated primary tumors, *n* = 89	High level of miR-210 expression was associated with a higher risk of recurrence than a lower level of miR-210	[50]	Target genes:*ACVR1B, CBFA2T1* Predicted pathways: cell cycle, cell adhesion and immune response
miR-26a	Frozen tamoxifen-treated tumors of metastatic patients, *n* = 235	Higher levels of miR-26a were significantly associated with clinical benefit (*i.e*., complete or partial response, or stable disease), and with favorable TTP (*i.e.*, first detection of disease progression)	[51]	Target genes: *CDC2*, *CCNE1* Predicted pathways: cell cycle regulation pathway
miR-126-5p and miR-10a-5p	FFPE from postmenopausal tamoxifen-treated patients, Validation set: *n* = 34 with recurrence; *n* = 47 without recurrence	Low expression of miR-10a and miR-126 correlated significantly with reduced relapse-free time	[47]	Target genes: n/a Predicted pathways: n/a
miR-519a-3p	GEO datasets of breast cancer patients, Discovery set: *n* = 632, Validation set: *n* = 586	High expression of miRNA-519a correlated significantly with poorer disease-free survival in ER^+^ breast cancer patients	[48]	Target genes: *PTEN*, *RB1*, *CDKN1A/p21* Predicted pathways: PI3K/Akt pathway

FFPE: Formalin-fixed and paraffin-embedded; GEO: Gene Expression Omnibus; PFS: progression-free survival; TTP: time to progression.

### 2.2. Ingenuity Pathway Analysis

To investigate the biological interactions of the seven tamoxifen-related microRNAs from our literature search with other molecules, we used *in silico* analysis to find predicted targets and identify their corresponding networks. The predicted targets and networks were generated through the use of QIAGEN’s Ingenuity Pathway Analysis (IPA^®^, QIAGEN, Redwood City, CA, USA). This software collects information about molecule-to-molecule interactions, biological networks and canonical pathways in the Ingenuity Knowledge database. This information is also reviewed by experts to ensure good quality information. Additionally, the software calculates a *p* value (right-tailed Fisher’s exact test) to determine the probability that the input genes are connected to a verified network or pathway by chance alone.

**Figure 4:**
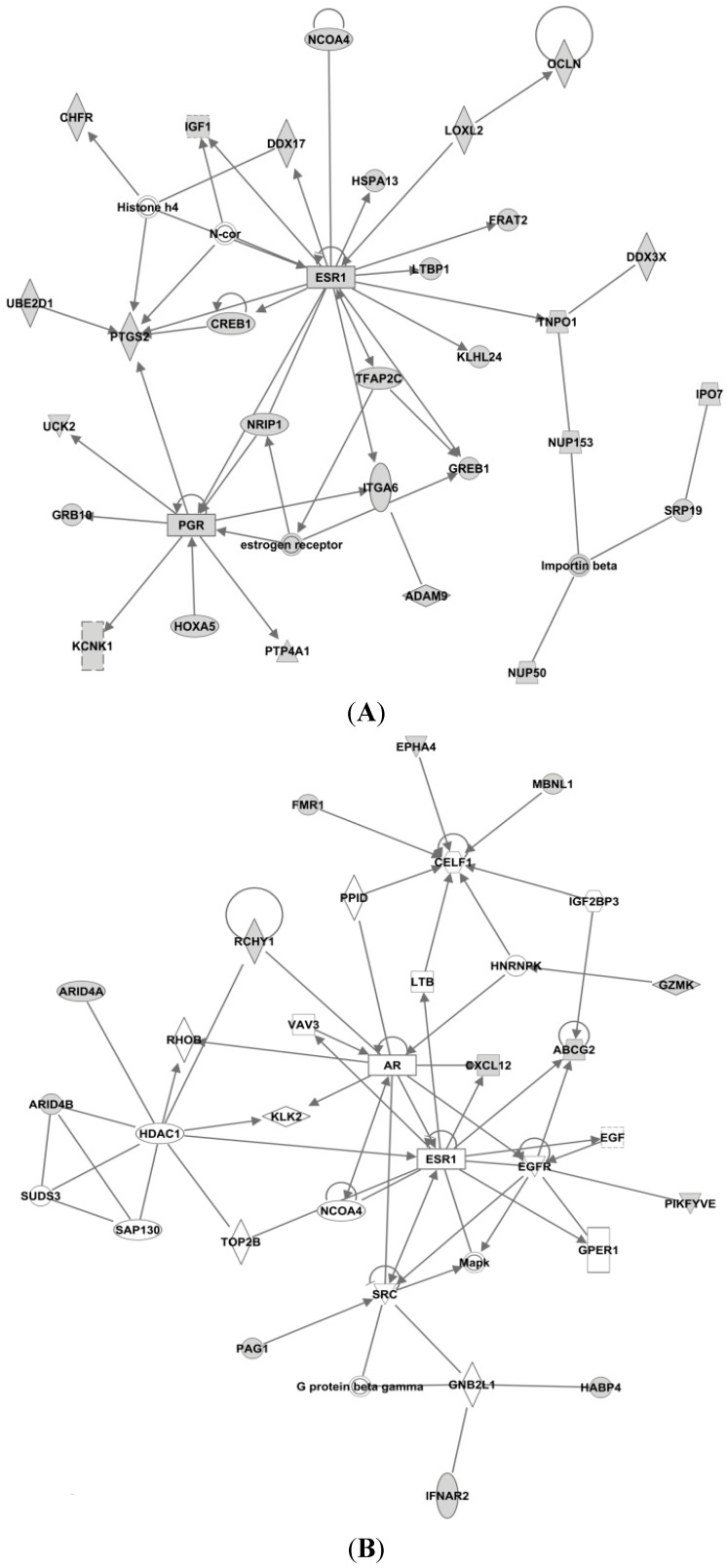

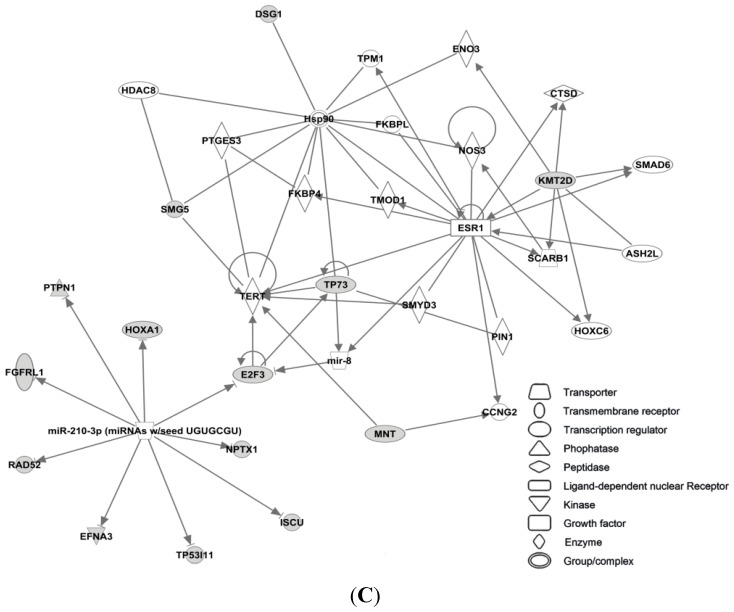
IPA networks for miR-26a, network 2 (**A**), miR-519a, network 1 (**B**) and miR-210, network 1 (**C**), centered on the estrogen receptor (ESR1). These networks created by IPA comprise networks with ER as a direct (miR-26a) or indirect (miR-519a and miR-210) target. Shaded boxes refer to direct targets whilst clear boxes refer to indirect targets of the specific miRNA.

**Figure 5:**
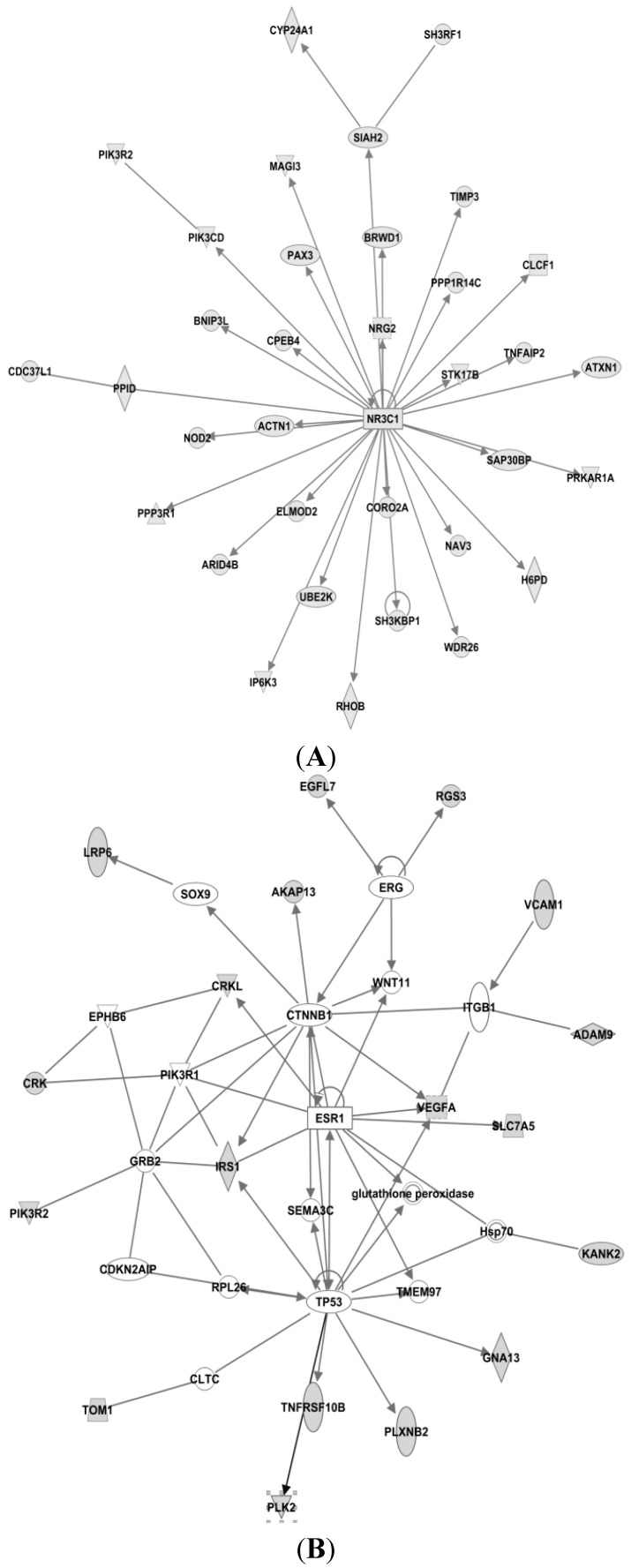

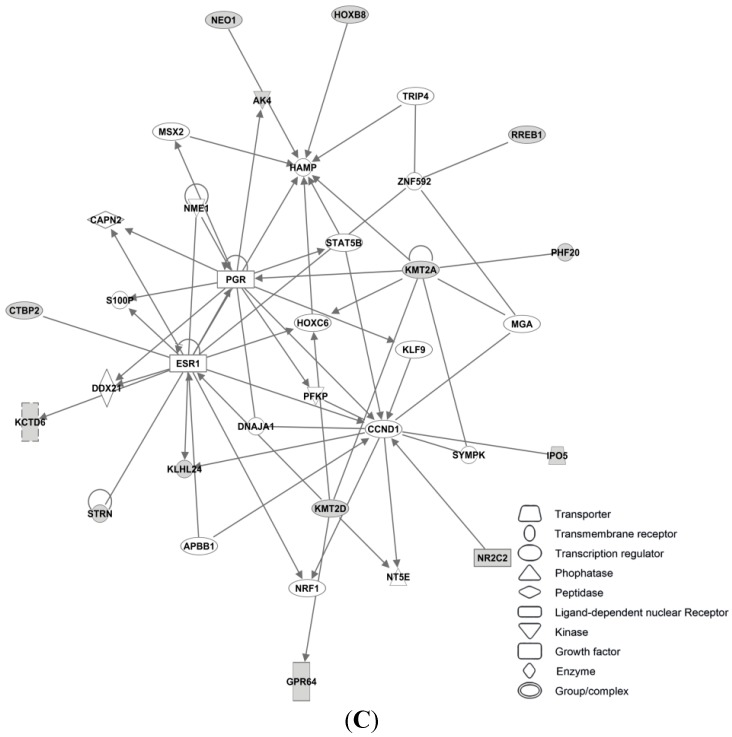
IPA networks for miR-30c, network (**A**), miR-126, network 1 (**B**) and miR-342, network 2 (**C**), centered on the estrogen receptor (ESR1). These networks created by IPA, comprise networks with ER as an indirect target (miR-126 and miR-342), whereas miR-30c has an ER-complex as an indirect target. Shaded boxes refer to direct targets whilst clear boxes refer to indirect targets of the specific miRNA.

**Figure 6:**
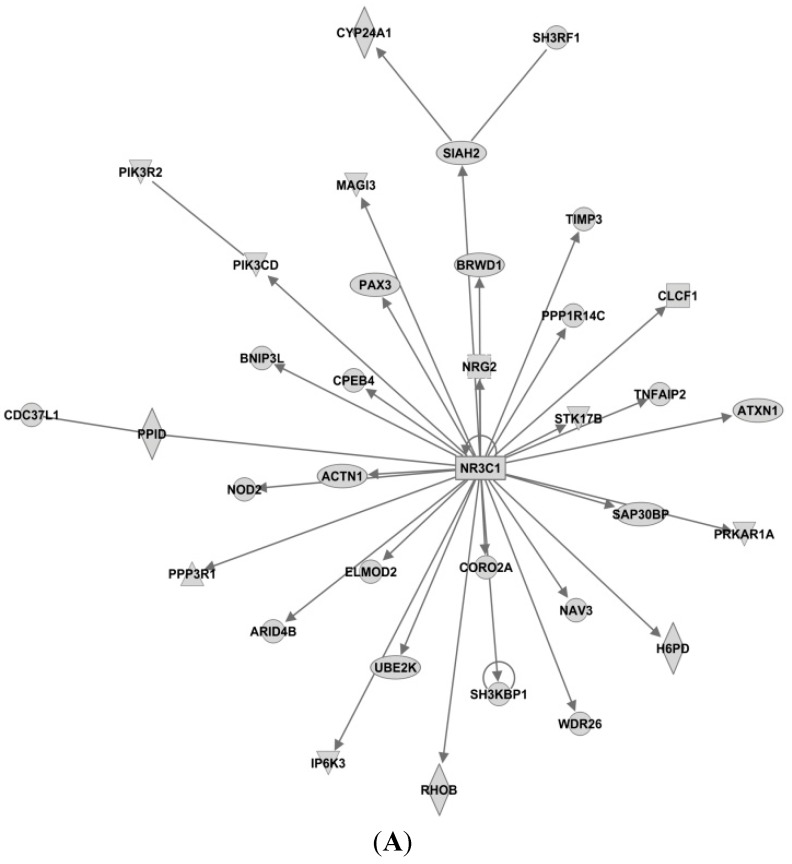

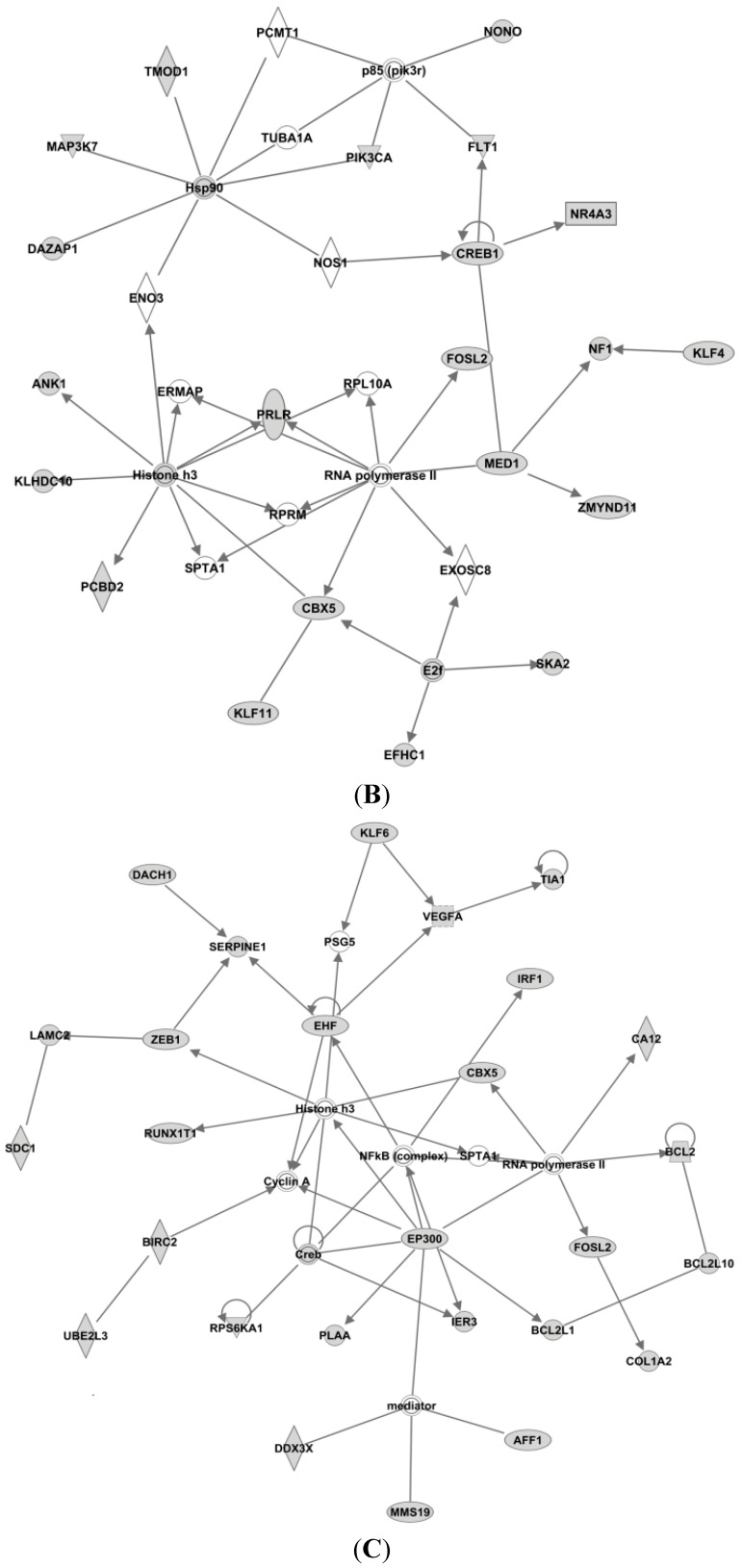

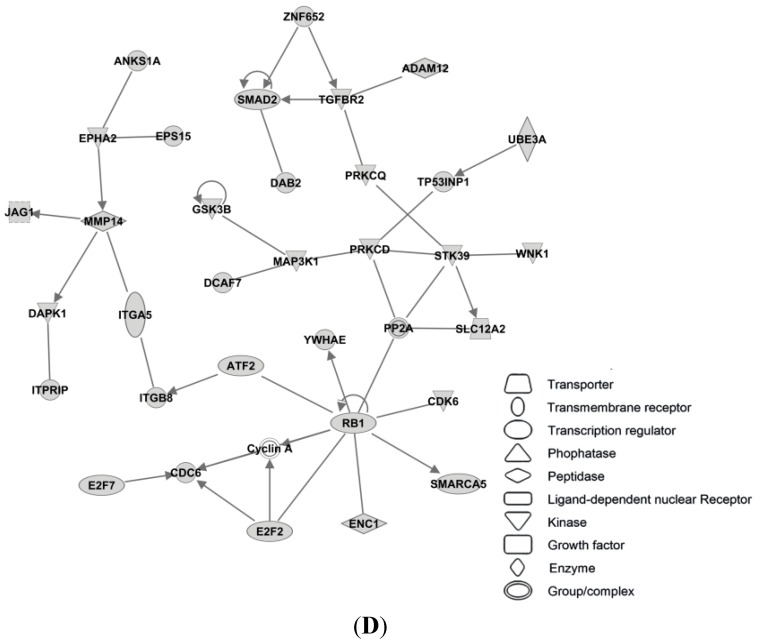
Top networks of miR-30c, network 1 (**A**), miR-10a, network 1 (**B**), miR-342, network 1 (**C**) and miR-26a, network 1 (**D**). Shown here are networks with the highest scores calculated by IPA, showing direct targets of the miRNAs. Shaded boxes refer to direct targets whilst clear boxes refer to indirect targets of the specific miRNA.

First, we used IPA to find the experimentally observed and highly predicted targets for each of the seven microRNAs, to be used further in an IPA core analysis (see Table 2). Then, for each of the resulting target lists, we used IPA to perform a core analysis considering only direct relationships between molecules, in humans, resulting in biological networks (see Figure 4, Figure 5 and Figure 6).

**Table 2:** Number of experimentally observed and highly predicted gene targets for the candidate microRNAs listed in Table 1.

MicroRNA	No. of Target Genes
miR-342-5p	337
miR-30c-5p	1420
miR-210-3p	78
miR-26a	892
miR-126a-5p	37
miR-10a-5p	338
miR-519a-3p	86

## 3. MicroRNAs in Breast Cancer

### 3.1. MicroRNAs in Breast Cancer Tumor Tissue

In breast cancer, several microRNAs are aberrantly expressed in tumor tissue compared to normal tissue. In a recent review, van Schooneveld lists some of the best described microRNAs in breast cancer including the oncogenic microRNAs miR-10b, -21, -155, -520c, -373, and the tumor suppressor microRNAs miR-31, -125b, -126, -200, -206, and -335 [52].

A well-known oncogenic microRNA is miR-21, which is overexpressed in breast cancer [53,54,55] and has been correlated with advanced stage, lymph node metastasis and poor prognosis [55]. Correspondingly, cell growth, migration and proliferation were inhibited when miR-21 was knocked down in MCF-7 and MDA-231 cells [56].

Among other well-studied microRNA clusters, the miR-17-92 cluster (comprising miR-17, miR-18a, miR-19a, miR-20a, miR-19b-1 and miR-92a-1) has been implicated in breast cancer by several studies. For example, in breast cancer cell lines, miR-17 has been shown to play an important role in promoting tumor cell migration and invasion [57]. In a study of ER+ breast cancer patients, ER has been shown to be a direct target of miR-18a and miR-18b [58], and miR-18a, together with miR-18b, has been associated with features of basal-like breast cancer [59]. Moreover, miR-17, miR-18a and miR-20a showed enhanced expression in triple-negative tumors compared to luminal A tumors [60]. In addition, miR-92a has been associated with tumor grade, cell migration and macrophage infiltration in breast cancer [61].

Studies report distinct functions of individual microRNAs, demonstrating that microRNAs have cell-, tissue- and organ-specific functions. As microRNAs appear to play important roles in breast cancer development, it seems likely that they have potential utility as prognostic and predictive biomarkers.

### 3.2. Circulating MicroRNAs in Breast Cancer

MicroRNAs have been detected in the circulation, either bound to lipids or proteins, inside apoptotic bodies from dead cells, or as part of circulating exosomes [62]. The presence of circulating microRNAs has also been shown in breast cancer patients. For instance, differential concentrations of miR-16, miR-107, miR-130a and miR-146a microRNAs were shown in plasma from 111 patients with different cancer subtypes [63]. In a cohort of 89 breast cancer patients (range 31–82 years), Roth *et al.* (2010) found elevated levels of circulating miR-10b, miR-155 and miR-34 in cell-free serum from breast cancer patients, compared with 29 healthy controls. The differences in relative concentrations of these microRNAs could be used to distinguish healthy controls from breast cancer patients, as well as metastatic (*n* = 30) from non-metastatic (*n* = 59) disease. Furthermore, in the 59 patients without distant metastases, higher levels of serum miR-34a correlated with advanced tumor stages [64]. In 2012, Madhavan *et al*. demonstrated a significant correlation between higher levels of eight circulating microRNAs and circulating tumor cells (CTC), in metastatic breast cancer patients (*n* = 133) compared with healthy controls (*n* = 76), thus showing the potential of circulating microRNAs as surrogate markers for CTCs. In addition, they found that miR-200b was a promising prognostic marker of both overall- and progression-free survival (PFS) [65].

Compared to intracellular microRNA or microRNA in cell-free blood (plasma or serum), exosomes have proved to be an enriched and protective source of circulating microRNAs [66,67]. Tumor cells secrete exosomes, so-called tumor-derived exosomes [68], in higher amounts than normal cells. In fact, the cargo of the tumor-derived exosomes has been shown to reflect the cell and tissue it originates from. This opens up the possibility to use tumor-derived exosomes detected in blood to gain information on the remaining tumor cells, providing a minimally invasive biomarker to detect tumor cells, which might also reveal some of the oncogenic features of the tumor.

The recent finding of microRNA in tumor-specific exosomes increases the potential for using microRNAs in blood as biomarkers for monitoring breast cancer characteristics and maybe even therapy response. Several studies have demonstrated the presence of tumor-derived exosomes containing microRNA, suggesting their potential as diagnostic, prognostic and predictive biomarkers.

Furthermore, exosomes originating from drug resistant breast cancer cells have been shown to mediate drug efflux and resistance through so-called exosomal shuttle-microRNAs [69]. In a recent study of chemo-resistant breast cancer cells (resistant to Adriamycin and Docetaxel), exosomes were shown to mediate such chemo-resistance to cells that were still sensitive to these drugs. This transfer of resistance was likely due to intercellular transfer of specific exosomal microRNAs, potentially miR-100, miR-222 and miR-30a [70]. Moreover, in another recent study of tamoxifen-sensitive and tamoxifen-resistant MCF-7 cells, exosomes released from resistant cells, were able to enter into tamoxifen-sensitive cells and release miR-221 and miR-222. These microRNAs then reduced the expression of p27 and ER in the recipient cells, thus decreasing their sensitivity to tamoxifen [71].

### 3.3. Tamoxifen-Related MicroRNAs Found in Breast Cancer Tissue

In 2010, Cittelly *et al.* showed that miR-342-5p was differentially expressed in tamoxifen-sensitive *versus* tamoxifen-resistant cell lines. MicroRNA-342 expression was shown to be suppressed in the tamoxifen-resistant breast cancer cells, while a miR-342 inhibitor could promote resistance in the tamoxifen-sensitive cells. In addition, 16 tamoxifen-treated primary breast tumors (*n* = 6 with recurrence, *n* = 10 without recurrence) were analyzed by *in situ* hybridization (ISH) for miR-342 expression. Due to the low sample number estimates were imprecise, but the ISH results indicated that the level of miR-342 expression was about two-fold higher in tumors from the tamoxifen responders (*i.e*., those without recurrence or metastasis), compared to the non-responders. Furthermore, they performed a search for potential gene targets by microarray analysis of tamoxifen resistant breast cancer cell lines with restored level of miR-342 and control cells. This microanalysis showed that 13 genes were differentially expressed, of which GEMIN4 and BMP7 were validated as direct targets of miR-342. By using Ingenuity Pathway Analysis (IPA) for identification of functional pathways enriched with miR-342 regulated genes, they identified the Cell Death and Apoptosis of Breast Cancer Cells pathways as being the most significant. By canonical pathway analysis, IPA identified Mitotic Roles of Polo-Like Kinase as the pathway in which miR-342 genes were most significantly enriched [46].

In another study of 246 ER+ advanced breast cancers, higher expression of miR-30a-3p, miR-30c and miR-182 was associated with better response to tamoxifen treatment as measured by longer progression-free survival time; however, only miR-30c was shown to be an independent predictor. These patients were initially hormone-naïve, and received tamoxifen treatment following metastases or recurrence. For some of the samples included in this study both microRNA and mRNA expression data was available and used to analyze the potential underlying biological pathways associating these microRNAs with tamoxifen resistance. Accordingly, by using Global Test/Biocarta, miR-30c was found to be significantly correlated to HER2, signal transduction, and oncology pathway, whereas genes related to miR-30a-3p expression were significantly associated with Ceramide signaling pathway. Furthermore, both miR-30c and miR-30a-3p were negatively associated with the RAC1 cell motility signaling pathway. By searching publicly available databases, they also reported PPARGC1B, Makorin-3, UBAC1 and PTPDC1 as target genes for both miR-30c and miR-30a [49].

In 89 ER+ tamoxifen-treated breast cancers, a higher risk of recurrence and poorer clinical outcome was associated with a high level of miR-210 expression, compared with low miR-210 expression. Overexpression of miR-210 in ER+ MCF7 cells, and repression in ER− MDA-MB-231 cells induced the altered expression of several genes (data not shown). Gene set enrichment analysis of these differentially expressed genes showed their involvement in biological pathways involved such as cell cycle, cell adhesion and immune response [50].

Low levels of Enhancer of Zeste homolog 2 (EZH2), a target of miR-26a, have been associated with favorable outcome in tamoxifen-treated patients [72]. In a retrospective study by Jansen *et al*. of 235 tamoxifen-treated patients with metastatic disease, high levels of miR-26a and decreased (EZH2) expression was associated with clinical benefit and favorable time to progression. Furthermore, pathway analysis on microarray data from 65 of these tumors using the Global Test Approach (GTA) indicated the cell cycle regulatory pathway and the gene CDC2 to be correlated with miR-26a expression [51]. In cell line models CDC2 has been linked to tamoxifen response [73]. Patients with lower mRNA levels of CDC2 also showed a delay in disease progression compared to those with higher levels [51].

In a retrospective study, miR-126 and miR-10a were reported as being independent predictors for tumor relapse in a study restricted to post-menopausal women with breast cancer following tamoxifen treatment. By microarray profiling, they screened 12 patients (matched on age at diagnosis, tumor size, grade, nodal status, PgR-and Her2/neu-status, ER immune reactive (IR) score and radiotherapy) with (*n* = 6) and without (*n* = 6) relapse following five years of tamoxifen treatment for 1105 microRNAs. Of the 20 resulting microRNAs, miR-126 and mir-10a were confirmed by qRT-PCR in a set of 81 patients with and without relapse [47].

More recently, Ward *et al*. described that the microRNA cluster C19MC (comprising 50 microRNAs) is upregulated in tamoxifen-resistant *versus* tamoxifen-sensitive breast cancer cells; miR-519a was the microRNA most highly correlated with tamoxifen resistance. Oncogenic miR-519a was also demonstrated to increase resistance to tamoxifen-induced apoptosis as well as cell viability and cell cycle progression. In addition, the oncogenic properties of miR-519a were confirmed in gene expression datasets (Gene Expression Omnibus; GEO) of breast cancer patients. Among patients who received tamoxifen, higher expression of miR-519a was correlated with poorer disease-free survival in patients with ER+ tumors. Furthermore, by using algorithms for microRNA predictive targets, they validated the tumor suppressor genes PTEN of the PI3K/Akt pathway, and retinoblastoma protein (RB1) and CDKN1A/p21 as direct targets of miR-519a [48].

### 3.4. Candidate MicroRNAs in Signaling Pathways and Their Relevant Target Genes in Tamoxifen Resistance

For each of the seven candidate microRNAs involved in patient tamoxifen response, our IPA analyses generated a list of predicted target genes (Table 2).

Based on these results, IPA generated several networks depicting various direct and indirect targets for each of the selected microRNAs (Figure 4, Figure 5 and Figure 6). The networks are graphically represented as explained in the legend in the figure.

#### 3.4.1. Targets of miR-26a

The IPA-analyses demonstrate that six of the seven microRNAs are directly or indirectly associated with estrogen receptor; *i.e.* miR-126, miR-210, miR-26a, miR-519a, miR-30c and miR-342. As seen in Figure 4A, only miR-26a is highly predicted to directly target the *ER gene* (synonymous to *ESR1* shown in Figure 4), while the other microRNAs are only indirectly linked to ESR1.

In the top network of miR-26a seen in Figure 6D, the well-known tumor suppressor protein retinoblastoma 1 (RB1) is predicted to be a direct target of miR-26a. As mentioned, Ward *et al*. identified RB1 to be targeted by miR-519a, but this is not mapped as a target in our IPA network for miR-519a. In 2007, deregulation of the RB1 pathway was shown to be associated with early recurrence following tamoxifen monotherapy [74]. Also, cyclin-dependent kinase 6 (CDK6) is a direct target of miR-26a.

As seen in Figure 4A, insulin-like growth factor 1 (IGF1) is also predicted as a direct target for miR-26a, this is very interesting as IGF1 is highly expressed in the presence of estradiol [75]. IGF1 binds to the IGF1 receptor (IGF1R) and activates downstream pathways such as mitogen-activated protein kinase (MAPK) and PI3K pathways [76]. IGF1R and ER are strongly connected [77], and because of their crosstalk, the combination of IGF1R and ER antagonists has been clinically tested; although without convincing results [78].

#### 3.4.2. Targets of miR-519

In Figure 4B for miR-519a, Phosphoprotein membrane anchor with glycosphingolipid microdomains 1 (PAG1) is seen as a direct target, while the proto-oncogene tyrosine-protein kinase Src is seen as an indirect target for miR-519a. Interestingly, by inhibiting the activity of Src in MCF7-cells, estrogen-stimulated proliferation was blocked [79]. Similarly, when constitutively active Src was expressed in endocrine-sensitive MCF-7 cells, the cells response to tamoxifen was attenuated, whereas tamoxifen-resistant MCF7-cells were re-sensitized when Src was suppressed. Additionally, elevated Src activity in tumor tissue was associated with clinically poor prognosis [80]. Furthermore, Src expression and Src-phosphorylation has been found to be increased in tamoxifen resistant T47D-cells. In the same study, membrane expression of Src on tamoxifen-treated breast tumor cells was associated with reduced disease-free and overall survival [81]. EGFR, MAPK and GPER1 are also indirect targets of miR-519a. GPER (G protein-coupled estrogen receptor) is inversely associated with tamoxifen resistance as confirmed in a cohort study of 103 patients [82]. Moreover, in a study by Yuan *et al*., GPER was shown to be important in the initiation/induction of tamoxifen resistance, and is thought to contribute to tamoxifen resistance by interaction with EGFR during long-term treatment with tamoxifen in breast cancer cells. This crosstalk leads to phosphorylation of MAPK and AKT thus stimulating ER-independent gene transcription and development of tamoxifen resistance [83].

#### 3.4.3. Targets of miR-210

Homeobox A1 (HOXA1) is predicted to act as a direct target for miR-210 (Figure 4C). The HOXA1 is an ER-regulated gene and the HOXA1 locus is believed to be involved in promoting growth of tamoxifen resistant breast cancer cells. ER forms a complex with lysine (K)-specific demethylase 3A (KDM3A), which indirectly regulates the transcriptional outcome of the HOXA1 locus. This results in increased activation of ER in the presence of tamoxifen [84,85]. Heat shock protein 90 (Hsp90) is another direct target of miR-210. Chaperone molecules, of which Hsp90 is one of the most common, are involved in many important cellular pathways, especially in regulating the folding and sorting of proteins, as well as in the cells response to stress, cellular homeostasis, and cell cycle control [86]. It has been shown that tamoxifen and its metabolite 4-OHT may enhance the ATPase activity of Hsp90. The active metabolite was identified as a putative ligand for Hsp90 [87]. More recently, inhibition of Hsp90 has been shown to dramatically impair the emergence of resistance to hormone antagonists (tamoxifen and fulvestrant) in both cell culture and mice [88].

#### 3.4.4. Targets of miR-30c

In network 3 of miR-30c in Figure 5A, an estrogen receptor complex was found as an indirect target of miR-30c. In addition, forkhead box A1 (FOXA1) is a predicted target of miR-30c in this network, and is associated with ER. FOXA1 is important in ER-binding to chromatin, and is shown to be important for ER functioning as well as endocrine response in breast cancer cells [89,90].

miR-30c had the highest number of predicted target genes (1420), and as presented in Figure 6A, all were direct targets. Among them are cytochrome P450, family 24, subfamily A, polypeptide 1 (CYP24A1), phosphatidylinositol-4,5-bisphosphate 3-kinase, catalytic subunit delta (PIK3CD), phosphoinositide-3-kinase regulatory subunit 2 (PIK3R2) and TIMP metallopeptidase inhibitor 3 (TIMP3). CYP24A1 is a member of the cytochrome P450 superfamily. This enzymatic family plays important roles in drug metabolism and the synthesis of steroids and cholesterol. CYP24A1 is involved in regulation of vitamin D3 level, calcium homeostasis and the vitamin D endocrine system [91]. In both tamoxifen-sensitive and -resistant breast cancer cells, 1α,25-dihydroxyvitamin D3 has an antiproliferative effect [92]. TIMP3 inhibits matrix metalloproteinases, and is seen as a direct target of miR-30c, but also a direct target for miR-221 and miR-222. Suppression of these microRNAs leads to an increased sensitivity to tamoxifen, mediated by TIMP3, in ER+ MCF-7 cells [93].

In addition, PIK3CD and PIK3R2 are also two direct targets of miR-30c. PIK3R2 is also seen as a direct target of miR-126 (see Figure 5A). Another such kinase, the phosphatidylinositol-4,5-bisphosphate 3-kinase (PIK3CA), is a predicted direct target gene of miR-10a. PIK3CD, PIK3R2 and PIK3CA are all members of the Class I phosphoinositide 3-kinase (PI3K) enzymes, which have been shown to be involved in several types of cancer and involved in the Akt/mTOR pathway [94,95]. PI3KCA mutations are frequent in breast cancer, especially in ER+ breast cancer. In fact, 40% of luminal breast cancers have PI3KCA mutations [96], making this the most common mutation in breast cancer. Furthermore, the overall mutation rate in the whole PI3K pathway in breast cancer is >70% [97]. Activation of the PI3K pathway can lead to activation of proliferation, or growth and inhibition of apoptosis. Even though PIK3CA mutations have a high mutation frequency in breast cancer patients, it does not seem to be a good independent predictor in the context of endocrine therapy [98].

#### 3.4.5. Targets of miR-126

As seen in Table 2, miR-126 had the fewest predicted targets genes (37) in our IPA analysis. In network 1 of miR-126 (Figure 5B), PI3KR2 is shown as a direct target, whereas Hsp 70, CLTC and TP53 are shown as indirect targets. Hsp70 is a component of the molecular chaperone machinery, which aids in assembly and trafficking of steroid receptors. Clathrin heavy chain (CLTC) is involved in intracellular trafficking as well as endocytosis, and was recently identified as a target of miR-574-3p, which again was shown to modulate tamoxifen-resistance in MCF-7 cells [99].

#### 3.4.6. Targets of miR-342

As seen in Figure 6C, the transcription factor Zinc Finger E-box Binding Homeobox (ZEB1) is a direct target of miR-342. ZEB1 has previously been associated with increased tamoxifen resistance and reduced expression of miR-200 in LY2 endocrine resistant breast cancer cells [100]. As illustrated in the network, another direct target of miR-342 is B-cell CLL/Lymphoma 2 (BCL2), an oncogene that is involved in regulation of apoptosis. In tamoxifen resistant cell line studies miR-15a and miR-16 have also been shown to activate BCL2 expression, and thereby promote resistance in HER2/ER^+^ breast cancer cells [101]. In Figure 5C both ESR1 and PGR are shown as indirect targets of miR-342. Homeobox B1 (HOXB1) is seen as a direct target.

#### 3.4.7. Targets of miR-10a

As seen in Figure 6A, apart from PIK3CA, miR-10a also targets heat-shock protein 90 (Hsp90) and Histone H3. Hsp90 is discussed previously, see Figure 4C. Histone H3 is part of the nucleosome and involved in transcription regulation and proliferation. Interestingly, miR-10a and miR-342 share five targets in their top rated networks; CBX5, FOSL2, Histone H3, RNA polymerase II and spectrin, alpha, erythrocytic 1 (SPTA1). Chromobox homolog 5 (CBX5) is a heterochromatin protein associated with centromeres. Spectrin alpha 1 (SPTA1) is a scaffold protein. FOS-Like antigen 2 (FOSL2) is a member of the Fos gene family that encodes leucine zipper proteins and have been implicated in cell proliferation, transformation and differentiation [91]. However, no correlations between these genes and tamoxifen resistance have been reported at this moment.

## 4. Discussion

**Figure 7:**
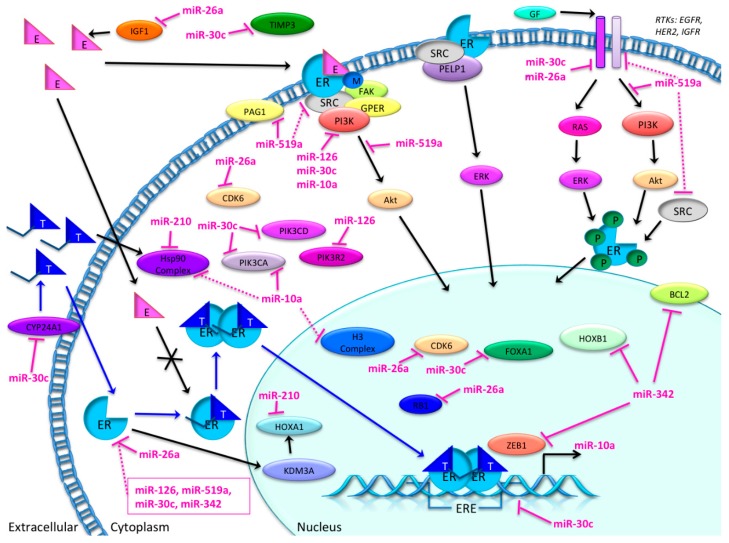
Overview of the possible involvement of candidate microRNAs in tamoxifen resistance pathways, based on the present literature search and IPA analysis. MicroRNAs miR-10a, -26a, -30c, -126, -210, -342-5p and -519a and their direct (pink lines) or indirect (dotted lines) targets. BCL2: B-cell CLL/lymphoma 2; CDK6: cyclin-dependent kinase 6; CYP24A1: cytochrome P450, family 24, subfamily A, polypeptide 1; IGF1: insulin-like growth factor 1; FOXA1: forkhead box A1; HOXA1: homeobox A1; HOXB1: homeobox B1; Hsp90: heat-shock protein 90. KDM3A: lysine (K)-specific demethylase 3A. PAG1: phosphoprotein membrane anchor with glycosphingolipid microdomains 1; PI3K: phosphoinositide 3-kinase; PIK3CA: phosphatidylinositol-4,5-bisphosphate 3-kinase, catalytic subunit alpha; PIK3CD: phosphatidylinositol-4,5-bisphosphate 3-kinase, catalytic subunit delta; PIK3R2: phosphoinositide-3-kinase, regulatory subunit 2 (beta); RB1: retinoblastoma 1; TIMP3: TIMP metallopeptidase inhibitor 3; ZEB1: zinc finger E-box binding homeobox 1. Black arrow: normal pathway. Blue arrow: tamoxifen pathway. Crossed arrow: disrupted pathway. Pink inhibition arrow: direct inhibition by miRNA. Dotted pink inhibition arrow: indirect inhibition by miRNA.

In the present paper, we review the potential of miRNAs as biomarkers for predicting the response to tamoxifen in breast cancer patients (Figure 7). Surprisingly, among more than 2400 known microRNAs, only seven have been associated with tamoxifen resistance according to our literature search, where we focused only on studies that included patient material. These seven microRNAs could well be part of the bigger picture, as many more microRNAs have been correlated with tamoxifen resistance from *in vitro* analyses.

Our IPA analysis suggests that signaling involved in endocrine resistance can be divided in two main signaling networks: One related to ER-signaling (Figure 4) and the second to membrane-related receptors (*i.e.*, EGFR, HER-2, IGF) (Figure 5). As observed in these predicted target networks and discussed before, tamoxifen resistance does not seem to be directly related to changes in ER itself, and, as Figure 5 implies, changes in various molecules in the network surrounding ER could contribute to tamoxifen resistance. These networks and pathways may have an important role in tamoxifen resistance, and therefore the role of these microRNAs should be further investigated. Notably, no link between microRNAs and tamoxifen metabolic pathways (e.g., CYP2D6) was found in our IPA analysis. This may reflect the negative results in studies examining the relation between genetic polymorphisms and relapses on tamoxifen treatment [102].

As an alternative explanation of endocrine resistance, the switch to other ER signaling independent pathways (e.g., the EGFR pathway) that drive the cellular survival processes are reported [36,103]. The IPA analyses also suggest that microRNAs related to signal networks independent of ER signaling might have a direct and strong connection to these pathways, indicating that these mechanisms are more important predictors of endocrine resistance than ER-related signaling networks.

The underlying heterogeneity of breast tumors is one important explanation of this observation [104]. In ER+ tumors (*i.e.*, ≥1% positively stained tumor cells by immunohistochemistry) most clones may respond to endocrine treatment, while others are non-responders. In the former type, up-regulation of various receptor tyrosine kinases (RTKs), *i.e.*, HER-2 may take place [105,106]. This phenomenon is thought to be an escape mechanism from endocrine control of cancer cells.

Examples of microRNAs involved in the switch to alternative ER-independent pathways are miR-10a, miR-126 and miR-30c relation to PI3K signaling. This pathway is a central node in mediating growth factor receptor signaling, and is known to characterize the more aggressive and less endocrine sensitive luminal B-subtype of breast cancers [107]. Studies indicate that only a slight loss in inhibition of this potent signaling pathway (*i.e.*, loss of PTEN) is enough to induce endocrine resistance. Interestingly, this escape from endocrine control can be restored by targeting mTOR, Protein Kinase B (Akt) or Mitogen Activated Kinase that are located downstream in the same pathway [108]. Moreover, lessons from the treatment of metastatic ER+ breast cancer patients support the importance of targeting RTKs to restore the endocrine sensitivity in breast cancer tissue. Reliable predictive markers for the PI3K/Akt/mTOR axis are necessary to indicate the need for co-targeting PI3K and ER pathways to restore endocrine sensibility. The importance of these microRNAs is demonstrated since they may help identify the timing of change in treatment strategy.

Interestingly, microRNAs are involved in various hallmarks of cancer and can interact with several characteristics at the same time [109]. From our list of microRNAs involved in tamoxifen resistance (Table 2), cell proliferation and invasion is enhanced by elevation of miR-210 [50,110], cell survival is promoted by a decrease in miR-26a [111], and angiogenesis is stimulated by elevation of miR-126 [112]. Multigene assays show that proliferation related genes dominate the basis of the predictive effect of chemotherapy in endocrine responsive early breast cancer [113,114]. However, molecular subtyping is suitable for short-term evaluation only (*i.e.*, the first five years of follow, as they seem to lose their power in long-term perspectives [115]).

Thus, since microRNAs interact with several hallmarks of cancer simultaneously, detailed characterization of these microRNAs may provide an improved understanding of the underlying resistance mechanism as compared to the currently established biomarkers in breast cancer. Promising predictors for late recurrences are involvement of micro-environmental stromal factors and the EMT processes [116]. Factors that can predict long-term cancer cell survival will be of particular interest since endocrine therapy is given over a long time span (Figure 2).

Clinicians face various challenges when deciding the most effective endocrine therapy for each patient. The following four clinical scenarios all have the need for reliable biomarkers that can point out which patients can be assigned tamoxifen treatment from those who cannot. First, in younger patients, tamoxifen is increasingly given as adjuvant therapy to patients operated for ductal carcinoma *in situ*. It increases event-free survival, even at the population level [117]; Second, in the younger pre-menopausal patients with invasive ER+ breast cancer, it is recommended to administer either 10 years with tamoxifen, or five years with OFS in addition to tamoxifen or AI (Figure 2). The latter regime is shown to be superior to tamoxifen monotherapy, but is not very well tolerated [118]; Third, in postmenopausal women AIs for five years upfront or for at least two years followed by tamoxifen for three years is recommended (Figure 2); Fourth, the elderly co-morbid patients are often treated with endocrine treatment only, while tumor size is under surveillance. Elderly women are especially vulnerable to AIs, e.g., due to high fracture rates [119]. Since the side effects of AIs are quite substantial and probably highly underestimated [120], biomarkers that can identify tamoxifen-responsive tumors, so that AIs can be avoided, are clinically relevant. Selection of the tamoxifen-sensitive tumors by means of reliable biomarkers would be clinically very helpful in managing these patients, also to indicate development of endocrine resistance so other treatment options can be considered (*i.e.*, radiation therapy or surgery). All these scenarios share the need to distinguish between tamoxifen and alternative regimens that comprise either OFS or an AI. Today, clinicians are not able to take into account the heterogeneity of the tumors; as a consequence, we treat breast cancer with a wide specter of ER positivity (*i.e.*, ranging from ≥1% up to 100%) with the same endocrine strategy.

The tumor heterogeneity calls for a double strategy to monitor endocrine effectiveness. During the early phase of the tamoxifen treatment, identification of possible endocrine sensitive/responsive tumors might provide important information whether or not endocrine therapy will work. Later, during long-term follow-up, markers that can identify emerging endocrine resistant clones at an early stage might allow change of therapy before the endocrine resistance becomes clinically evident. This “dual-approach” is more in line with the intrinsic tumor biology of breast cancer. In the large adjuvant ATAC [34] and BIG-1 98 trials [35], tamoxifen was compared with anastrozole and letrozole, respectively. These studies provide valuable long-term follow-up data on relapse and survival in four different treatment arms. In order to get an indication of which microRNA might depict endocrine sensitivity in the adjuvant setting, microRNA profiling of patients from these two studies could promptly provide valuable information on this issue. Such studies would also provide microRNA profiles from treatment arms that switch from tamoxifen to AI and vice versa. Moreover, microRNA analyses in population-based epidemiological studies (e.g., our ongoing work) will strengthen the findings from clinical trials. Candidate microRNAs identified should be validated in other prospective trials. Such prospective studies should comprise a combinatory analysis of microRNA expression profiles in the primary tumor together with concurrent free microRNAs in serum or plasma. Moreover, patients enrolled in neo-adjuvant treatment trials and endocrine-controlled comorbid elderly patients, are especially suitable for repeated tumor biopsies during treatment. Repeated microRNA profiling of both primary tumors and plasma microRNAs during treatment will elucidate which microRNAs might be promising marker candidates; both for endocrine sensitivity (if the tumor shrinks) as well as endocrine resistance (if the tumor stops responding or increases in size).

## 5. Conclusions

In conclusion, endocrine treatment remains one of the most important strategies for eradicating and/or controlling ER positive breast cancer. Biomarkers of endocrine responsiveness and resistance are urgently needed to help clinicians in making treatment decisions. As microRNAs are able to exert control at the translational level, they are an important link between coding genes and the various cellular processes. Hence, microRNAs are certainly promising candidate biomarkers that could be used in the clinic to guide tamoxifen treatment. One immediate aim must be to include a combined microRNA profiling in tumor tissues and in plasma in on-going and future clinical studies with long-term follow-up. This approach seems like a small step for the role of microRNA as a biomarker, but will undoubtedly bring the clinical knowledge of microRNA in endocrine treatment in breast cancer a giant leap forward.

## References

[1] Haynes B.P., Viale G., Galimberti V., Rotmensz N., Gibelli B., A'Hern R., Smith I.E., Dowsett M. (2013). Expression of key estrogen-regulated genes differs substantially across the menstrual cycle in estrogen receptor-positive primary breast cancer. Breast Cancer Res. Treat..

[2] Baak J.P.A., van Diest P.J., Voorhorst F.J., van der Wall E., Beex L.V.A.M., Vermorken J.B., Janssen E.A.M., Gudlaugsson E., other collaborators of the Multicenter Morphometric Mammary Carcinoma Project (MMMCP) (2007). The prognostic value of proliferation in lymph-node-negative breast cancer patients is age dependent. Eur. J. Cancer.

[3] Goldhirsch A., Winer E.P., Coates A.S., Gelber R.D., Piccart-Gebhart M., Thurlimann B., Senn H.J. (2013). Personalizing the treatment of women with early breast cancer: Highlights of the St gallen international expert consensus on the primary therapy of early breast cancer 2013. Ann. Oncol..

[4] Cuzick J., Dowsett M., Pineda S., Wale C., Salter J., Quinn E., Zabaglo L., Mallon E., Green A.R., Ellis I.O. (2011). Prognostic value of a combined estrogen receptor, progesterone receptor, ki-67, and human epidermal growth factor receptor 2 immunohistochemical score and comparison with the genomic health recurrence score in early breast cancer. J. Clin. Oncol..

[5] National Comprehensive Cancer Network (NCCN). http://www.nccn.org/.

[6] Early Breast Cancer Trialists’ Collaborative Group (1998). Tamoxifen for early breast cancer: An overview of the randomised trials. Early breast cancer trialists’ collaborative group. Lancet.

[7] Early Breast Cancer Trialists’ Collaborative Group (2005). Effects of chemotherapy and hormonal therapy for early breast cancer on recurrence and 15-year survival: An overview of the randomised trials. Lancet.

[8] Carlson R.W., Allred D.C., Anderson B.O., Burstein H.J., Carter W.B., Edge S.B., Erban J.K., Farrar W.B., Forero A., Giordano S.H. (2011). Invasive breast cancer, clinical practice guidelines, NCCN. J. Natl. Compr. Cancer Netw..

[9] Burstein H.J., Prestrud A.A., Seidenfeld J., Anderson H., Buchholz T.A., Davidson N.E., Gelmon K.E., Giordano S.H., Hudis C.A., Malin J. (2010). American society of clinical oncology clinical practice guideline: Update on adjuvant endocrine therapy for women with hormone receptor-positive breast cancer. J. Clin. Oncol..

[10] Gnant M., Harbeck N., Thomssen C. (2011). St. Gallen 2011: Summary of the consensus discussion. Breast Care.

[11] Musgrove E.A., Sutherland R.L. (2009). Biological determinants of endocrine resistance in breast cancer. Nat. Rev. Cancer.

[12] Dos Anjos Pultz B., da Luz F.A., de Faria P.R., Oliveira A.P., de Araujo R.A., Silva M.J. (2014). Far beyond the usual biomarkers in breast cancer: A review. J. Cancer.

[13] Guo H., Ingolia N.T., Weissman J.S., Bartel D.P. (2010). Mammalian micrornas predominantly act to decrease target mrna levels. Nature.

[14] Kuiper G.G., Carlsson B., Grandien K., Enmark E., Haggblad J., Nilsson S., Gustafsson J.A. (1997). Comparison of the ligand binding specificity and transcript tissue distribution of estrogen receptors alpha and beta. Endocrinology.

[15] Paech K., Webb P., Kuiper G.G., Nilsson S., Gustafsson J., Kushner P.J., Scanlan T.S. (1997). Differential ligand activation of estrogen receptors eralpha and erbeta at ap1 sites. Science.

[16] Russo J., Russo I.H. (2004). Genotoxicity of steroidal estrogens. Trends Endocrinol. Metab..

[17] Tsai M.J., O’Malley B.W. (1994). Molecular mechanisms of action of steroid/thyroid receptor superfamily members. Annu. Rev. Biochem..

[18] Davies C., Pan H., Godwin J., Gray R., Arriagada R., Raina V., Abraham M., Alencar V.H.M., Badran A., Bonfill X. (2013). Long-term effects of continuing adjuvant tamoxifen to 10 years *versus* stopping at 5 years after diagnosis of estrogen receptor-positive breast cancer: Atlas, a randomised trial. Lancet.

[19] Hayes E.L., Lewis-Wambi J.S. (2015). Mechanisms of endocrine resistance in breast cancer: An overview of the proposed roles of noncoding RNA. Breast Cancer Res..

[20] Fisher B., Costantino J.P., Wickerham D.L., Cecchini R.S., Cronin W.M., Robidoux A., Bevers T.B., Kavanah M.T., Atkins J.N., Margolese R.G. (2005). Tamoxifen for the prevention of breast cancer: Current status of the national surgical adjuvant breast and bowel project p-1 study. J. Natl. Cancer Inst..

[21] Ellis P.A., Saccani-Jotti G., Clarke R., Johnston S.R., Anderson E., Howell A., A’Hern R., Salter J., Detre S., Nicholson R. (1997). Induction of apoptosis by tamoxifen and ici 182780 in primary breast cancer. Int. J. Cancer.

[22] Deligdisch L., Kalir T., Cohen C.J., de Latour M., Le Bouedec G., Penault-Llorca F. (2000). Endometrial histopathology in 700 patients treated with tamoxifen for breast cancer. Gynecol. Oncol..

[23] Heldring N., Pike A., Andersson S., Matthews J., Cheng G., Hartman J., Tujague M., Strom A., Treuter E., Warner M. (2007). Estrogen receptors: How do they signal and what are their targets. Physiol. Rev..

[24] Norwegian Breast Cancer Group (NBCG). http://nbcg.no/.

[25] Nass N., Kalinski T. (2015). Tamoxifen resistance: From cell culture experiments towards novel biomarkers. Pathology.

[26] Hoskins J.M., Carey L.A., McLeod H.L. (2009). Cyp2d6 and tamoxifen: DNA matters in breast cancer. Nat. Rev. Cancer.

[27] Srinivasan M., Sedmak D., Jewell S. (2002). Effect of fixatives and tissue processing on the content and integrity of nucleic acids. Am. J. Pathol..

[28] Gjerde J., Geisler J., Lundgren S., Ekse D., Varhaug J.E., Mellgren G., Steen V.M., Lien E.A. (2010). Associations between tamoxifen, estrogens, and fsh serum levels during steady state tamoxifen treatment of postmenopausal women with breast cancer. BMC Cancer.

[29] Cronin-Fenton D.P., Damkier P., Lash T.L. (2014). Metabolism and transport of tamoxifen in relation to its effectiveness: New perspectives on an ongoing controversy. Future Oncol..

[30] Lien E.A., Soiland H., Lundgren S., Aas T., Steen V.M., Mellgren G., Gjerde J. (2013). Serum concentrations of tamoxifen and its metabolites increase with age during steady-state treatment. Breast Cancer Res. Treat..

[31] Geisler J., Lonning P.E. (2005). Aromatase inhibition: Translation into a successful therapeutic approach. Clin. Cancer Res..

[32] Dowsett M., Jones A., Johnston S.R., Jacobs S., Trunet P., Smith I.E. (1995). *In vivo* measurement of aromatase inhibition by letrozole (cgs 20267) in postmenopausal patients with breast cancer. Clin. Cancer Res..

[33] Geisler J., Haynes B., Anker G., Dowsett M., Lonning P.E. (2002). Influence of letrozole and anastrozole on total body aromatization and plasma estrogen levels in postmenopausal breast cancer patients evaluated in a randomized, cross-over study. J. Clin. Oncol..

[34] Forbes J.F., Cuzick J., Buzdar A., Howell A., Tobias J.S., Baum M. (2008). Effect of anastrozole and tamoxifen as adjuvant treatment for early-stage breast cancer: 100-month analysis of the atac trial. Lancet Oncol..

[35] Mouridsen H., Giobbie-Hurder A., Goldhirsch A., Thurlimann B., Paridaens R., Smith I., Mauriac L., Forbes J., Price K.N., Regan M.M. (2009). Letrozole therapy alone or in sequence with tamoxifen in women with breast cancer. N. Engl. J. Med..

[36] Osborne C.K., Schiff R. (2011). Mechanisms of endocrine resistance in breast cancer. Annu. Rev. Med..

[37] Viedma-Rodriguez R., Baiza-Gutman L., Salamanca-Gomez F., Diaz-Zaragoza M., Martinez-Hernandez G., Ruiz Esparza-Garrido R., Velazquez-Flores M.A., Arenas-Aranda D. (2014). Mechanisms associated with resistance to tamoxifen in estrogen receptor-positive breast cancer (review). Oncol. Rep..

[38] Garcia-Becerra R., Santos N., Diaz L., Camacho J. (2012). Mechanisms of resistance to endocrine therapy in breast cancer: Focus on signaling pathways, mirnas and genetically based resistance. Int. J. Mol. Sci..

[39] Miller W.R. (2004). Biological rationale for endocrine therapy in breast cancer. Best Pract. Res. Clin. Endocrinol. Metab..

[40] Mirbase: The Microrna Database. http://www.mirbase.org/index.shtml.

[41] Griffiths-Jones S., Grocock R.J., van Dongen S., Bateman A., Enright A.J. (2006). Mirbase: Microrna sequences, targets and gene nomenclature. Nucleic Acids Res..

[42] Kozomara A., Griffiths-Jones S. (2014). Mirbase: Annotating high confidence micrornas using deep sequencing data. Nucleic Acids Res..

[43] Calin G.A., Dumitru C.D., Shimizu M., Bichi R., Zupo S., Noch E., Aldler H., Rattan S., Keating M., Rai K. (2002). Frequent deletions and down-regulation of micro- rna genes mir15 and mir16 at 13q14 in chronic lymphocytic leukemia. Proc. Natl. Acad. Sci. USA.

[44] Calin G.A., Sevignani C., Dumitru C.D., Hyslop T., Noch E., Yendamuri S., Shimizu M., Rattan S., Bullrich F., Negrini M. (2004). Human microrna genes are frequently located at fragile sites and genomic regions involved in cancers. Proc. Natl. Acad. Sci. USA.

[45] Zhang B., Pan X., Cobb G.P., Anderson T.A. (2007). Micrornas as oncogenes and tumor suppressors. Dev. Biol..

[46] Cittelly D.M., Das P.M., Spoelstra N.S., Edgerton S.M., Richer J.K., Thor A.D., Jones F.E. (2010). Downregulation of mir-342 is associated with tamoxifen resistant breast tumors. Mol. Cancer.

[47] Hoppe R., Achinger-Kawecka J., Winter S., Fritz P., Lo W.Y., Schroth W., Brauch H. (2013). Increased expression of mir-126 and mir-10a predict prolonged relapse-free time of primary estrogen receptor-positive breast cancer following tamoxifen treatment. Eur. J. Cancer.

[48] Ward A., Shukla K., Balwierz A., Soons Z., Konig R., Sahin O., Wiemann S. (2014). Microrna-519a is a novel oncomir conferring tamoxifen resistance by targeting a network of tumour-suppressor genes in ER+ breast cancer. J. Pathol..

[49] Rodriguez-Gonzalez F.G., Sieuwerts A.M., Smid M., Look M.P., Meijer-van Gelder M.E., de Weerd V., Sleijfer S., Martens J.W., Foekens J.A. (2011). Microrna-30c expression level is an independent predictor of clinical benefit of endocrine therapy in advanced estrogen receptor positive breast cancer. Breast Cancer Res. Treat..

[50] Rothe F., Ignatiadis M., Chaboteaux C., Haibe-Kains B., Kheddoumi N., Majjaj S., Badran B., Fayyad-Kazan H., Desmedt C., Harris A.L. (2011). Global microrna expression profiling identifies mir-210 associated with tumor proliferation, invasion and poor clinical outcome in breast cancer. PLoS ONE.

[51] Jansen M.P., Reijm E.A., Sieuwerts A.M., Ruigrok-Ritstier K., Look M.P., Rodriguez-Gonzalez F.G., Heine A.A., Martens J.W., Sleijfer S., Foekens J.A. (2012). High mir-26a and low cdc2 levels associate with decreased ezh2 expression and with favorable outcome on tamoxifen in metastatic breast cancer. Breast Cancer Res. Treat..

[52] Van Schooneveld E., Wildiers H., Vergote I., Vermeulen P.B., Dirix L.Y., van Laere S.J. (2015). Dysregulation of micrornas in breast cancer and their potential role as prognostic and predictive biomarkers in patient management. Breast Cancer Res..

[53] Iorio M.V., Ferracin M., Liu C.G., Veronese A., Spizzo R., Sabbioni S., Magri E., Pedriali M., Fabbri M., Campiglio M. (2005). Microrna gene expression deregulation in human breast cancer. Cancer Res..

[54] Sempere L.F., Christensen M., Silahtaroglu A., Bak M., Heath C.V., Schwartz G., Wells W., Kauppinen S., Cole C.N. (2007). Altered microrna expression confined to specific epithelial cell subpopulations in breast cancer. Cancer Res..

[55] Yan L.X., Huang X.F., Shao Q., Huang M.Y., Deng L., Wu Q.L., Zeng Y.X., Shao J.Y. (2008). Microrna mir-21 overexpression in human breast cancer is associated with advanced clinical stage, lymph node metastasis and patient poor prognosis. RNA.

[56] Yan L.X., Wu Q.N., Zhang Y., Li Y.Y., Liao D.Z., Hou J.H., Fu J., Zeng M.S., Yun J.P., Wu Q.L. (2011). Knockdown of mir-21 in human breast cancer cell lines inhibits proliferation, *in vitro* migration and *in vivo* tumor growth. Breast Cancer Res..

[57] Li H., Bian C., Liao L., Li J., Zhao R.C. (2011). Mir-17–5p promotes human breast cancer cell migration and invasion through suppression of hbp1. Breast Cancer Res. Treat..

[58] Leivonen S.K., Makela R., Ostling P., Kohonen P., Haapa-Paananen S., Kleivi K., Enerly E., Aakula A., Hellstrom K., Sahlberg N. (2009). Protein lysate microarray analysis to identify micrornas regulating estrogen receptor signaling in breast cancer cell lines. Oncogene.

[59] Jonsdottir K., Janssen S.R., Da Rosa F.C., Gudlaugsson E., Skaland I., Baak J.P., Janssen E.A. (2012). Validation of expression patterns for nine mirnas in 204 lymph-node negative breast cancers. PLoS ONE.

[60] Calvano Filho C.M., Calvano-Mendes D.C., Carvalho K.C., Maciel G.A., Ricci M.D., Torres A.P., Filassi J.R., Baracat E.C. (2014). Triple-negative and luminal a breast tumors: Differential expression of mir-18a-5p, mir-17–5p, and mir-20a-5p. Tumour Biol..

[61] Nilsson S., Moller C., Jirstrom K., Lee A., Busch S., Lamb R., Landberg G. (2012). Downregulation of mir-92a is associated with aggressive breast cancer features and increased tumour macrophage infiltration. PLoS ONE.

[62] Hunter M.P., Ismail N., Zhang X., Aguda B.D., Lee E.J., Yu L., Xiao T., Schafer J., Lee M.L., Schmittgen T.D. (2008). Detection of microrna expression in human peripheral blood microvesicles. PLoS ONE.

[63] Stuckrath I., Rack B., Janni W., Jager B., Pantel K., Schwarzenbach H. (2015). Aberrant plasma levels of circulating mir-16, mir-107, mir-130a and mir-146a are associated with lymph node metastasis and receptor status of breast cancer patients. Oncotarget.

[64] Roth C., Rack B., Muller V., Janni W., Pantel K., Schwarzenbach H. (2010). Circulating microRNAs as blood-based markers for patients with primary and metastatic breast cancer. Breast Cancer Res..

[65] Madhavan D., Zucknick M., Wallwiener M., Cuk K., Modugno C., Scharpff M., Schott S., Heil J., Turchinovich A., Yang R. (2012). Circulating mirnas as surrogate markers for circulating tumor cells and prognostic markers in metastatic breast cancer. Clin. Cancer Res..

[66] Cheng L., Sharples R.A., Scicluna B.J., Hill A.F. (2014). Exosomes provide a protective and enriched source of mirna for biomarker profiling compared to intracellular and cell-free blood. J. Extracell. Vesicles.

[67] Kosaka N., Yoshioka Y., Hagiwara K., Tominaga N., Ochiya T. (2013). Functional analysis of exosomal microrna in cell-cell communication research. Methods Mol. Biol..

[68] Taylor D.D., Black P.H. (1986). Shedding of plasma membrane fragments. Neoplastic and developmental importance. Dev. Biol..

[69] Zhao L., Liu W., Xiao J., Cao B. (2015). The role of exosomes and “exosomal shuttle microrna” in tumorigenesis and drug resistance. Cancer Lett..

[70] Chen W.X., Liu X.M., Lv M.M., Chen L., Zhao J.H., Zhong S.L., Ji M.H., Hu Q., Luo Z., Wu J.Z. (2014). Exosomes from drug-resistant breast cancer cells transmit chemoresistance by a horizontal transfer of micrornas. PLoS ONE.

[71] Wei Y., Lai X., Yu S., Chen S., Ma Y., Zhang Y., Li H., Zhu X., Yao L., Zhang J. (2014). Exosomal miR-221/222 enhances tamoxifen resistance in recipient ER-positive breast cancer cells. Breast Cancer Res. Treat..

[72] Reijm E.A., Jansen M.P., Ruigrok-Ritstier K., van Staveren I.L., Look M.P., van Gelder M.E., Sieuwerts A.M., Sleijfer S., Foekens J.A., Berns E.M. (2011). Decreased expression of ezh2 is associated with upregulation of ER and favorable outcome to tamoxifen in advanced breast cancer. Breast Cancer Res. Treat..

[73] Johnson N., Bentley J., Wang L.Z., Newell D.R., Robson C.N., Shapiro G.I., Curtin N.J. (2010). Pre-clinical evaluation of cyclin-dependent kinase 2 and 1 inhibition in anti-estrogen-sensitive and resistant breast cancer cells. Br. J. Cancer.

[74] Bosco E.E., Wang Y., Xu H., Zilfou J.T., Knudsen K.E., Aronow B.J., Lowe S.W., Knudsen E.S. (2007). The retinoblastoma tumor suppressor modifies the therapeutic response of breast cancer. J. Clin. Investig..

[75] Dupont J., Le Roith D. (2001). Insulin-like growth factor 1 and oestradiol promote cell proliferation of mcf-7 breast cancer cells: New insights into their synergistic effects. Mol. Pathol..

[76] Riedemann J., Macaulay V.M. (2006). Igf1r signalling and its inhibition. Endocr. Relat. Cancer.

[77] Fagan D.H., Yee D. (2008). Crosstalk between igf1r and estrogen receptor signaling in breast cancer. J. Mammary Gland Biol. Neoplasia.

[78] Chen H.X., Sharon E. (2013). Igf-1r as an anti-cancer target—Trials and tribulations. Chin. J. Cancer.

[79] Planas-Silva M.D., Hamilton K.N. (2007). Targeting c-Src kinase enhances tamoxifen’s inhibitory effect on cell growth by modulating expression of cell cycle and survival proteins. Cancer Chemother. Pharmacol..

[80] Morgan L., Gee J., Pumford S., Farrow L., Finlay P., Robertson J., Ellis I., Kawakatsu H., Nicholson R., Hiscox S. (2009). Elevated src kinase activity attenuates tamoxifen response *in vitro* and is associated with poor prognosis clinically. Cancer Biol. Ther..

[81] Larsen S.L., Laenkholm A.V., Duun-Henriksen A.K., Bak M., Lykkesfeldt A.E., Kirkegaard T. (2015). Src drives growth of antiestrogen resistant breast cancer cell lines and is a marker for reduced benefit of tamoxifen treatment. PLoS ONE.

[82] Ignatov A., Ignatov T., Weissenborn C., Eggemann H., Bischoff J., Semczuk A., Roessner A., Costa S.D., Kalinski T. (2011). G-protein-coupled estrogen receptor gpr30 and tamoxifen resistance in breast cancer. Breast Cancer Res. Treat..

[83] Yuan J., Liu M., Yang L., Tu G., Zhu Q., Chen M., Cheng H., Luo H., Fu W., Li Z. (2015). Acquisition of epithelial-mesenchymal transition phenotype in the tamoxifen-resistant breast cancer cell: A new role for g protein-coupled estrogen receptor in mediating tamoxifen resistance through cancer-associated fibroblast-derived fibronectin and beta1-integrin signaling pathway in tumor cells. Breast Cancer Res..

[84] Mahajan K., Lawrence H.R., Lawrence N.J., Mahajan N.P. (2014). Ack1 tyrosine kinase interacts with histone demethylase kdm3a to regulate the mammary tumor oncogene hoxa1. J. Biol. Chem..

[85] Mahajan K., Mahajan N.P. (2014). Ack1/tnk2 tyrosine kinase: Molecular signaling and evolving role in cancers. Oncogene.

[86] Lianos G.D., Alexiou G.A., Mangano A., Mangano A., Rausei S., Boni L., Dionigi G., Roukos D.H. (2015). The role of heat shock proteins in cancer. Cancer Lett..

[87] Zhao R., Leung E., Gruner S., Schapira M., Houry W.A. (2010). Tamoxifen enhances the hsp90 molecular chaperone atpase activity. PLoS ONE.

[88] Whitesell L., Santagata S., Mendillo M.L., Lin N.U., Proia D.A., Lindquist S. (2014). Hsp90 empowers evolution of resistance to hormonal therapy in human breast cancer models. Proc. Natl. Acad. Sci. USA.

[89] Hurtado A., Holmes K.A., Ross-Innes C.S., Schmidt D., Carroll J.S. (2011). Foxa1 is a critical determinant of estrogen receptor function and endocrine response. Nat. Genet..

[90] Ross-Innes C.S., Stark R., Teschendorff A.E., Holmes K.A., Ali H.R., Dunning M.J., Brown G.D., Gojis O., Ellis I.O., Green A.R. (2012). Differential oestrogen receptor binding is associated with clinical outcome in breast cancer. Nature.

[91] Pruitt K.D., Brown G.R., Hiatt S.M., Thibaud-Nissen F., Astashyn A., Ermolaeva O., Farrell C.M., Hart J., Landrum M.J., McGarvey K.M. (2014). Refseq: An update on mammalian reference sequences. Nucleic Acids Res..

[92] Lundqvist J., Yde C.W., Lykkesfeldt A.E. (2014). 1alpha,25-dihydroxyvitamin d3 inhibits cell growth and nfkappab signaling in tamoxifen-resistant breast cancer cells. Steroids.

[93] Gan R., Yang Y., Yang X., Zhao L., Lu J., Meng Q.H. (2014). Downregulation of mir-221/222 enhances sensitivity of breast cancer cells to tamoxifen through upregulation of timp3. Cancer Gene Ther..

[94] Zhao L., Vogt P.K. (2008). Class i pi3k in oncogenic cellular transformation. Oncogene.

[95] Liu P., Cheng H., Roberts T.M., Zhao J.J. (2009). Targeting the phosphoinositide 3-kinase (pi3k) pathway in cancer. Nat. Rev. Drug Discov..

[96] Sabine V.S., Crozier C., Brookes C.L., Drake C., Piper T., van de Velde C.J., Hasenburg A., Kieback D.G., Markopoulos C., Dirix L. (2014). Mutational analysis of pi3k/akt signaling pathway in tamoxifen exemestane adjuvant multinational pathology study. J. Clin. Oncol..

[97] Miller T.W., Rexer B.N., Garrett J.T., Arteaga C.L. (2011). Mutations in the phosphatidylinositol 3-kinase pathway: Role in tumor progression and therapeutic implications in breast cancer. Breast Cancer Res..

[98] Juric D., Castel P., Griffith M., Griffith O.L., Won H.H., Ellis H., Ebbesen S.H., Ainscough B.J., Ramu A., Iyer G. (2015). Convergent loss of pten leads to clinical resistance to a pi(3)kalpha inhibitor. Nature.

[99] Ujihira T., Ikeda K., Suzuki T., Yamaga R., Sato W., Horie-Inoue K., Shigekawa T., Osaki A., Saeki T., Okamoto K. (2015). Microrna-574–3p, identified by microrna library-based functional screening, modulates tamoxifen response in breast cancer. Sci. Rep..

[100] Manavalan T.T., Teng Y., Litchfield L.M., Muluhngwi P., Al-Rayyan N., Klinge C.M. (2013). Reduced expression of mir-200 family members contributes to antiestrogen resistance in ly2 human breast cancer cells. PLoS ONE.

[101] Cittelly D.M., Das P.M., Salvo V.A., Fonseca J.P., Burow M.E., Jones F.E. (2010). Oncogenic her2 delta 16 suppresses mir-15a/16 and deregulates bcl-2 to promote endocrine resistance of breast tumors. Carcinogenesis.

[102] Lash T.L., Lien E.A., Sorensen H.T., Hamilton-Dutoit S. (2009). Genotype-guided tamoxifen therapy: Time to pause for reflection?. Lancet Oncol..

[103] Lonning P.E. (2010). Molecular basis for therapy resistance. Mol. Oncol..

[104] Russnes H.G., Navin N., Hicks J., Borresen-Dale A.L. (2011). Insight into the heterogeneity of breast cancer through next-generation sequencing. J. Clin. Investig..

[105] Flageng M.H., Moi L.L., Dixon J.M., Geisler J., Lien E.A., Miller W.R., Lonning P.E., Mellgren G. (2009). Nuclear receptor co-activators and her-2/neu are upregulated in breast cancer patients during neo-adjuvant treatment with aromatase inhibitors. Br. J. Cancer.

[106] Moi L.L., Flageng M.H., Gjerde J., Madsen A., Rost T.H., Gudbrandsen O.A., Lien E.A., Mellgren G. (2012). Steroid receptor coactivators, her-2 and her-3 expression is stimulated by tamoxifen treatment in dmba-induced breast cancer. BMC Cancer.

[107] Creighton C.J., Fu X., Hennessy B.T., Casa A.J., Zhang Y., Gonzalez-Angulo A.M., Lluch A., Gray J.W., Brown P.H., Hilsenbeck S.G. (2010). Proteomic and transcriptomic profiling reveals a link between the pi3k pathway and lower estrogen-receptor (ER) levels and activity in ER+ breast cancer. Breast Cancer Res..

[108] Fu X., Creighton C.J., Biswal N.C., Kumar V., Shea M., Herrera S., Contreras A., Gutierrez C., Wang T., Nanda S. (2014). Overcoming endocrine resistance due to reduced pten levels in estrogen receptor-positive breast cancer by co-targeting mammalian target of rapamycin, protein kinase b, or mitogen-activated protein kinase kinase. Breast Cancer Res..

[109] Goh J.N., Loo S.Y., Datta A., Siveen K.S., Yap W.N., Cai W., Shin E.M., Wang C., Kim J.E., Chan M. (2015). Micrornas in breast cancer: Regulatory roles governing the hallmarks of cancer. Biol. Rev. Camb. Philos. Soc..

[110] Volinia S., Galasso M., Sana M.E., Wise T.F., Palatini J., Huebner K., Croce C.M. (2012). Breast cancer signatures for invasiveness and prognosis defined by deep sequencing of microRNA. Proc. Natl. Acad. Sci. USA.

[111] Tan S., Ding K., Li R., Zhang W., Li G., Kong X., Qian P., Lobie P.E., Zhu T. (2014). Identification of mir-26 as a key mediator of estrogen stimulated cell proliferation by targeting chd1, greb1 and kpna2. Breast Cancer Res..

[112] Zhang J., Du Y.Y., Lin Y.F., Chen Y.T., Yang L., Wang H.J., Ma D. (2008). The cell growth suppressor, mir-126, targets irs-1. Biochem. Biophys. Res. Commun..

[113] Paik S., Shak S., Tang G., Kim C., Baker J., Cronin M., Baehner F.L., Walker M.G., Watson D., Park T. (2004). A multigene assay to predict recurrence of tamoxifen-treated, node-negative breast cancer. N. Engl. J. Med..

[114] Albain K.S., Paik S., van’t Veer L. (2009). Prediction of adjuvant chemotherapy benefit in endocrine responsive, early breast cancer using multigene assays. Breast.

[115] Engstrom M.J., Opdahl S., Hagen A.I., Romundstad P.R., Akslen L.A., Haugen O.A., Vatten L.J., Bofin A.M. (2013). Molecular subtypes, histopathological grade and survival in a historic cohort of breast cancer patients. Breast Cancer Res. Treat..

[116] Cheng Q., Chang J.T., Gwin W.R., Zhu J., Ambs S., Geradts J., Lyerly H.K. (2014). A signature of epithelial-mesenchymal plasticity and stromal activation in primary tumor modulates late recurrence in breast cancer independent of disease subtype. Breast Cancer Res..

[117] Lo A.C., Truong P.T., Wai E.S., Nichol A., Weir L., Speers C., Hayes M.M., Baliski C., Tyldesley S. (2015). Population-based analysis of the impact and generalizability of the nsabp-b24 study on endocrine therapy for patients with ductal carcinoma *in situ* of the breastdagger. Ann. Oncol..

[118] Bernhard J., Luo W., Ribi K., Colleoni M., Burstein H.J., Tondini C., Pinotti G., Spazzapan S., Ruhstaller T., Puglisi F. (2015). Patient-reported outcomes with adjuvant exemestane *versus* tamoxifen in premenopausal women with early breast cancer undergoing ovarian suppression (text and soft): A combined analysis of two phase 3 randomised trials. Lancet Oncol..

[119] Soiland H., Hagen K.B., Gjerde J., Lende T.H., Lien E.A. (2013). Breaking away: High fracture rates may merit a new trial of adjuvant endocrine therapy in scandinavian breast cancer patients. Acta Oncol..

[120] Lintermans A., Neven P. (2015). Safety of aromatase inhibitor therapy in breast cancer. Expert Opin. Drug Saf..

